# Cocoa (*Theobroma cacao* L.) Seed-Derived Peptides Reduce Blood Pressure by Interacting with the Catalytic Site of the Angiotensin-Converting Enzyme

**DOI:** 10.3390/foods10102340

**Published:** 2021-09-30

**Authors:** Luis Jorge Coronado-Cáceres, Blanca Hernández-Ledesma, Luis Mojica, Lucía Quevedo-Corona, Griselda Rabadán-Chávez, Gustavo Adolfo Castillo-Herrera, Eugenia Lugo Cervantes

**Affiliations:** 1 Unidad de Tecnología Alimentaria, Centro de Investigación y Asistencia en Tecnología y Diseño del Estado de Jalisco, Guadalajara 44270, Mexico; lucoronado@ciatej.mx (L.J.C.-C.); lmojica@ciatej.mx (L.M.); gris.rab@gmail.com (G.R.-C.); gcastillo@ciatej.mx (G.A.C.-H.); 2 Instituto de Investigación en Ciencias de la Alimentación (CIAL, CSIC-UAM, CEI UAM+CSIC), 28049 Madrid, Spain; 3 Departamento de Fisiología, Escuela Nacional de Ciencias Biológicas, Instituto Politécnico Nacional, Wilfrido Massieu s/n Esq. Manuel I. Stampa, Col. Unidad Profesional Adolfo López Mateos, Mexico 07738, Mexico; quevedocorona@hotmail.com

**Keywords:** antihypertensive, angiotensin-converting enzyme, proteins, bioactive peptides, molecular docking, *Theobroma cacao* L.

## Abstract

This study aimed at determining the effect of cocoa proteins (CP) on the blood pressure, using in silico, in vitro and in vivo approaches. The in silico assay showed 26 Criollo cocoa peptides with alignment in the Blast^®^ analysis. Peptide sequences ranged from 6 to 16 amino acids, with molecular weight ranging from 560.31 to 1548.76 Da. The peptide sequences LSPGGAAV, TSVSGAGGPGAGR, and TLGNPAAAGPF showed the highest theoretical affinity with −8.6, −5.0, and −10.2 kcal/mol, for the angiotensin-converting enzyme (ACE), renin, and angiotensin II type 1 receptor (AT_1_-R), respectively. The Criollo CP hydrolysates (CPH) presented in vitro ACE inhibitory activity with an IC_50_ value of 0.49 mg/mL. Furthermore, the orogastric administration of 150 mg CP/kg/day in rats fed a high-fat (HF) diet (HF + CP group) showed a significant decrease in systolic blood pressure (SBP) by 5% (*p* < 0.001) and diastolic blood pressure (DBP) by 7% (*p* < 0.001) compared with the HF group. The human equivalent dose (HED) of CP for an adult (60 kg) is 1.45 g per day. These results suggest that the consumption of CP could reduce blood pressure by blocking ACE, and could be used as an ingredient in the elaboration of antihypertensive functional foods.

## 1. Introduction

According to the World Health Organization (WHO), in 2016, almost 2 billion adults were overweight and 600 million were obese. Obesity is a risk factor for developing non-communicable diseases (NCDs) such as cardiovascular disease, type 2 diabetes mellitus, neurodegenerative diseases and cancer [1]. NCDs are the leading cause of mortality worldwide, with an estimated 39.5 million deaths per year (72.3% of total death in 2016). The largest number of deaths for NCDs was caused by cardiovascular diseases (17.6 million). Global, deaths from cardiovascular disease increased by 14.5% between 2006 and 2016 [2].

Hypertension affects around 1.4 billion people globally [3,4] and represents the principal risk factor for ischemic heart disease and cerebrovascular disease (stroke). Ischemic heart disease and stroke accounted for more than 85% of cardiovascular disease deaths. The blood pressure is regulated by the renin-angiotensin system (RAS), integrated by enzymes renin and the angiotensin-converting enzyme (ACE). The renin is a 37 kDa protein that hydrolyse a 55 kDa hepatic protein (angiotensinogen) to produce an inactive decapeptide called angiotensin I (DRVYIHPFHL) [5]. ACE acts converting angiotensin I into angiotensin II (a potent vasoconstrictor) by removing the C-terminal dipeptide HL [5]. The inhibition of renin and ACE in the RAS pathway is considered an important therapeutic strategy in treating hypertension [3,6] which is a controllable risk factor for cardiovascular diseases. ACE is the molecular target of antihypertensive drugs such as Captopril, Lisinopril, Benazepril, and Zofenopril [7,8,9]. However, because of the side effects associated with the use of these drugs, the interest has focused on the search of antihypertensive compounds from natural sources [10]. Among these compounds, those from proteinaceous nature are the most promising. Peptides from different food sources such as sesame and hemp seeds, corn, spinach, egg white proteins, rapeseed, meat, flaxseed, insects, birdseed, and amaranth seed have been characterised by their antihypertensive properties [11,12,13]. 

The Criollo cocoa (*Theobroma cacao* L.) is a native plant to Central America, mainly Mexico’s southern and southeastern regions [14]. The cocoa seeds used for the manufacture of chocolate are of great economic importance worldwide [15]. The polyphenols and flavonoids of cocoa (PFC) have been considered the main responsible for the blood pressure reducing effects associated with cocoa consumption. The impact of PFC has been observed in both in vitro [16], and in vivo assays [17,18]. However, the existing data on the effect of cocoa proteins (CP) against hypertension is limited. Autolysates of Malaysian cocoa (unknown variety) have been demonstrated to inhibit ACE, analyzed by an in vitro assay [19]. However, to date, no animal models have been carried out to confirm the in vivo effects of CP. Thus, the objective of this work was to determine the effect of CP on blood pressure in an obesity-induced by diet rat model, and to elucidate the potential mechanism of action using in silico and in vitro models.

## 2. Materials and Methods

### 2.1. Materials

Cocoa seeds of the Criollo variety called “Carmelo” were obtained from the Municipality of Tuxtla Chico (Chiapas, Mexico). Six-week-old male Wistar rats (180 ± 5 g of body weight) were acquired from the Animal House of Autonomous Metropolitan University, Xochimilco Campus (Ciudad de Mexico, Mexico). Membrane was from Biotech CE 0.1–0.5 kDa MWCO (131060; Spectrum^TM^ Laboratories, Inc., Rancho Dominguez, CA, USA). ACEI Activity Assay Fluorometric Kit (CS0002) was purchased from Sigma-Aldrich^TM^, (St. Louis, MO, USA). 

### 2.2. Protein Extraction from Theobroma cacao L.

The coat and the mucilage of pods-derived seeds were removed, seeds were lyophilized and ground, and the flour was defatted in three phases: flour was dissolved in hexane:chloroform (2:1, *v*/*v*) at ratio 1:15 (*w*/*v*), magnetically stirred for 90 min, and centrifuged at 4700× *g* for 20 min at 4 °C [20]. The pellet was collected and dried in the extraction hood. The dry pellet was used to obtain the acetone dry powder (AcDP) [21]. AcDP was prepared to prevent polyphenols from disturbing analysis and avoiding the generation of false positives. The polyphenols were extracted three times with an 80% (*v*/*v*) cold aqueous acetone containing 5 mM sodium ascorbate; subsequently, with 70% (*v*/*v*) cold aqueous acetone; and finally, the residual water was removed from acetone pellet by dehydration with 100% cold acetone [21,22]. Each time, 20 min-magnetic stirring at 4 °C followed by centrifugation (15,000× *g* for 15 min at 4 °C) were carried out.

The CP were extracted following the protocols described by Voigt and coworkers [21] and Preza and coworkers [22], with some modifications, using three magnetic stirring steps (20 min at 4 °C) with the following buffers at a ratio of 1:10 (*w*/*v*) to extract the different protein fractions: albumins (water-soluble) (10 mM Tris-HCl containing 2 mM EDTA, pH 7.5); globulins (salt-soluble) (0.5 M NaCl containing 2 mM EDTA and 10 mM Tris-HCl, pH 7.5); prolamins (aqueous isopropanol-soluble); and glutenins (alkali-proteins) (0.1 N NaOH). After each step, samples were centrifuged at 15,000× *g* for 15 min at 4 °C, and supernatants were precipitated using 6 N HCl (pH 3.4) followed by centrifugation at 20,000× *g* for 20 min at 4 °C. The collected pellet was lyophilized for further uses. The protein concentration of CP was determined by the Lowry method using bovine serum albumin (BSA) as standard [23]. The protein content of the sample was 81%.

### 2.3. Sodium Dodecyl Sulfate-Polyacrylamide Gel Electrophoresis (SDS-PAGE)

Electrophoresis (SDS-PAGE) was performed in the Mini-PROTEAN system (Bio-Rad, Hercules, CA, USA), using 4% polyacrylamide stacking gels and 12.0% polyacrylamide resolving gels in reducing conditions and standard Tris-glycine buffers [24]. The image was obtained using a Gel Doc Documentation System (Gel Doc™ XR+ System, Bio-Rad Laboratories Inc., Hercules, CA, USA).

### 2.4. Simulated Gastrointestinal Digestion

The procedure described by Mojica and coworkers [25] was followed to simulate gastrointestinal digestion of CP. Briefly, the sequential digestion of CP (dissolved in water at a ratio 1:20 *w*/*v*) was performed with pepsin (enzyme/substrate ratio of 1:20 (*w*/*w*), pH 2.0, 2 h) and pancreatin (enzyme /substrate ratio of 1:20 (*w*/*w*), pH 7.5, 2 h) at 37 °C. The enzymes were inactivated by heating at 75 °C for 20 min, and the resulting CP hydrolyzates (CPH) were centrifuged at 20,000× *g* for 15 min at 4 °C, dialyzed using 100 to 500 Da molecular weight cut off membranes, freeze-dried, and stored at −20 °C until analysis.

### 2.5. Characterization of the Peptides and Sequence Identification

The freeze-dried CPH were analyzed by liquid chromatography (LC) coupled to tandem mass spectrometry (LC-ESI-MS/MS), using the methodology of Souza Rocha [26] with some modifications, a Q-tof Ultima mass spectrometer 175 (Waters Corp., Milford, MA, USA), equipped with an Alliance ^®^ 2795 HPLC system (Waters Corp.). Elution was performed by using a mobile phase composed of solvent A (95% H_2_O, 5% acetonitrile ACN, and 0.1% formic acid) and solvent B (95% ACN, 5% H_2_O, and 0.1% formic acid) at a flow rate of 400 µL/min. The linear gradient used was 0 min, 100% A; 2 min, 90% A; 6 min, 60% A; 10 min, 0% A; 12 min, 0% A; 12.10 min, 100% A; 15 min, 100% A, and the temperature of the analysis was 20 °C. Using a positive ion electrospray mode (+ESI), the analysis on the Q-tof was carried out in V-mode with an instrument resolution ranged from 9000 to 10,000 based on full width at half maximum, with a flow rate of 20 µL/min. The source and desolvation temperatures were set at 80 °C and 250 °C, respectively. The Q-tof was operated at a capillary voltage of 3.5 kV and a cone voltage of 35 V. The final detector was a microchannel plate with high sensitivity. The control of the instruments and the data processing was carried out with the MassLynx 4.1V software (Waters Corp.). Confirmation of peptides sequence was performed using the BLAST^®^ tool (http://www.blast.ncbi.nlm.nih.gov/Blast.cgi, accessed on 24 March 2021) [27].

### 2.6. Biological Potential and Molecular Docking of Peptides from T. cocoa

The biological potential of cocoa peptides was studied employing the BIOPEP-UWM database (http://www.uwm.edu.pl/biochemia/index.php/pl/biopep, accessed on 25 March 2021) [28]. The in silico biological potential of peptide sequences was evaluated based on the occurrence frequency of the fragments (amino acids) defined with the equation:

A = [a/N];

where “a” is the number of fragments with given activity in a sequence; “N” is the number of amino acid residues of protein/peptide.

Using the following steps: in the BIOPEP-UWM database portal; select “Bioactive Peptides”; “ANALYSIS”; “CALCULATIONS”; “FOR YOUR SEQUENCE”; Paste the sequence; “Report”. The peptides were designed and modeled using the 3D MarvinSketch program (Version 17.10, 2017, ChemAxon Lts, Záhony, Budapest, Hungary). 

The crystal structures of human ACE-complex (PDB: 1O8A) [29], renin (PDB: ID 2V0Z), and AT_1_-R (PDB: ID 4YAY) were acquired from the Protein Data Bank (http://www.rcsb.org/, accessed on 29 March 2021). Molecular docking methodology was used following Pan and Cao protocol [30] with some modifications. The best-ranked docking pose of the peptide in the active site of ACE, renin, and AT_1_-R was obtained according to the scores and binding-energy value. Hydrogen atoms, solvation parameters, and atomic charge were added with the help of AutoDockTools vina 4.5 [31], with a grid of 30 × 30 × 30 Å, and a radius of 1 Å. Peptide–enzyme interactions were revised, and the potential type of interactions was shown with the Discovery Studio 2019 Client viewer (Accelrys Software Inc., San Diego, CA, USA).

### 2.7. Angiotensin-Converting Enzyme (ACE) Inhibitory Activity

The ACEI Activity Assay test contained a synthetic fluorogenic peptide as the substrate, and the resulting fluorescence was proportional to ACE activity [32,33]. Briefly, all reagents were diluted in the assay buffer, according to the manufacturer’s instructions. A volume of 10 µL of assay buffer (control) or CPH (at six different concentrations prepared in assay buffer) was mixed with 40 µL of ACE. Subsequently, 50 µL of fluorogenic substrate warmed to 37 °C were added to experimental, control and blank sample wells, and the reaction was carried out at 37 °C [32]. ACE inhibitory activity was measured using a fluorescence plate reader (Tecan Infinite M200 PRO, Salzburg, Austria), with excitation and emission wavelengths of 320 and 405 nm, respectively. The % ACE inhibition was calculated using the formula:

ACE inhibitory activity (%) = 100 × [((FC − FB) − (FS − FBs))/(FC − FB)]

where FC (Control): Fluorescence emitted after the action of ACE on the substrate in the absence of inhibitor; FS (Sample): Fluorescence emitted after the action of ACE on the substrate in the presence of inhibitor sample. FB (Blank): Fluorescence emitted by the substrate. FBs (Blank sample): Fluorescence emitted by the substrate and the sample [34]. The IC_50_ was determined GraphPad Prism version 8.0.0 (GraphPad Software, San Diego, California USA, www.graphpad.com, accessed on 5–9 April 2021) by nonlinear regression of ACE inhibition (%) caused by six different concentrations of CPH.

### 2.8. Animal Assay

Obesity-induced six-week-old male Wistar rats were maintained under controlled conditions of humidity (40–60%) and temperature (22 ± 2 °C), with 12 h dark/12 h light cycles. The rats were acclimatized for one week with unlimited access to food and water. A total of 21 rats were used and randomly divided into three dietary groups (*n* = 7 per group) as follows [20,35]: Standard Diet (STD group, 3.1 kcal/g) (TD.05230; Teklad Global Harlan Laboratories, Inc., Madison, WI, USA), High-Fat diet (HF group, 4.5 kcal/g) (TD.88137; Teklad Global Harlan Laboratories, Inc.), and HF-diet, supplemented with 150 mg CP/kg/day through orogastric administration once daily (HF + CP group). The experimental period was 8 weeks. All animals were fed ad libitum with free access to water during the experimental period (8 weeks). The use of male Wistar rats was approved by the Ethics and Research Committee of the National School of Biological Sciences of the National Polytechnic Institute from Mexico [18,20,35,36].

### 2.9. Blood Pressure Measurement

Blood pressure measurements were carried out at a temperature of 36 °C in a noise-free environment by the tail-cuff method, using a computerized blood pressure system (IITC; Life Science Instruments, Woodland Hills, CA, USA). Three readings were taken consecutively on conscious rats at day 0, and after eight weeks of treatment, and the average was calculated and taken as a final reading for systolic blood pressure (SBP), diastolic blood pressure (DBP), mean blood pressure (MBP), and heart rate (HR) [18,36].

### 2.10. Calculation of Human Equivalent Doses (HED)

The animal dose should not be extrapolated to a human equivalent dose (HED) by a simple conversion based on body weight. For the more appropriate conversion of drug doses from animal to human studies, it is suggested the use of the Body Surface Area (BSA) normalization method [37] using the formula:

HED (mg/kg) = Animal doses (mg/kg) × [Animal K_m_ (6 for rat)/Human K_m_ (37 for human of 60 kg)]

where the K_m_ factor is body weight (kg) divided by BSA (m^2^), and it is used to convert the mg/kg dose used in a study to an mg/m^2^ dose. Values-based on data from the Food and Drug Administration (FDA) [37,38].

### 2.11. Statistical Analysis

Data are expressed as mean values ± standard error of the mean (SEM). All data were tested for normality and equality of variance using the Shapiro-Wilks and Levene’s tests. One-way ANOVA was conducted, followed by the Holm–Sidak test (SigmaPlot 12.0 from SYSTAT Software, San Jose, CA, USA) for multiple comparisons in all quantitative variables. A value of *p* < 0.05 represented a significant difference. Figure constructions were performed using GraphPad Prism ver. 6.01 (GraphPad Software, San Diego, CA, USA) [20].

## 3. Results

### 3.1. Protein Fractions and Identification of Criollo cocoa Peptides

**Figure 1:**
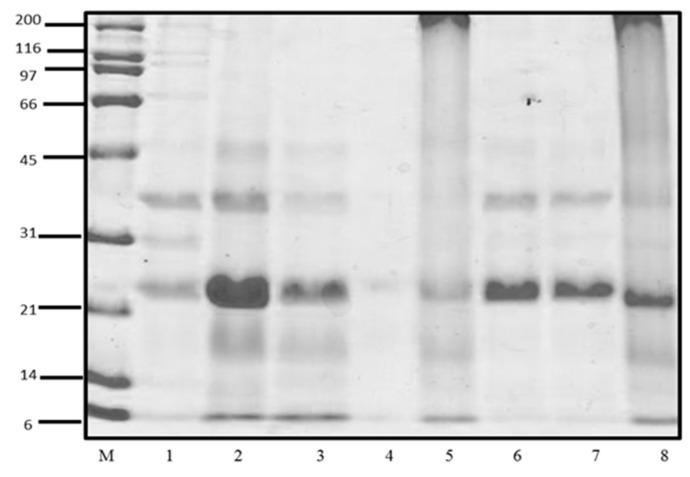
SDS-PAGE profiles of cacao protein (CP) fractions. M): molecular weight standard; 1: Albumin (Previous AcDP); 2: Albumins (AcDP); 3: Globulins (AcDP); 4: Prolamines (AcDP); 5: Glutelins (AcDP); 6: Total protein (pH 8.0); 7: Total protein (10 mM Tris pH, 7.5); 8: Total protein (0.1 M NaOH).

The study of the CP profiles was performed SDS-PAGE. In Figure 1, the profiles of soluble CP were observed, corresponding to the fractions of water-soluble protein (albumin), salt-soluble protein (globulin or vicilin), isopropanol-soluble protein (prolamin), and alkali-soluble protein (glutelin). The concentration of soluble proteins of Criollo cocoa is shown in Table 1. The fractions with the highest protein concentration were glutelins > albumin > globulins, while the low concentration was found in the prolamine fraction that was not used for the following assays.

Once collected, the water-soluble criollo CP were hydrolyzed and sequenced. In the chromatographic analysis, 55 peptide sequences were obtained, of which 26 were matched with the BLAST analysis. The peptide sequences presented molecular weights ranging from 560.31 to 1548.76 Da. Additionally, the biological potential of the cocoa peptide sequences was simulated using the BIOPEP-UWM database. The most common activities found were the dipeptidyl peptidase IV (DPP-IV) inhibitory activity related to diabetes, and the ACE and renin inhibitory activity and antithrombotic effects associated with cardiovascular diseases. Lower frequency of matched peptides corresponded to DPP-III inhibitory, antioxidant, chemotactic, anti-amnestic, and alpha-glucosidase inhibitory activities (Table 2).

### 3.2. Inhibitory Effect of Cocoa Peptides on RAS Enzymes by In Silico and In Vitro Analysis

**Figure 2:**
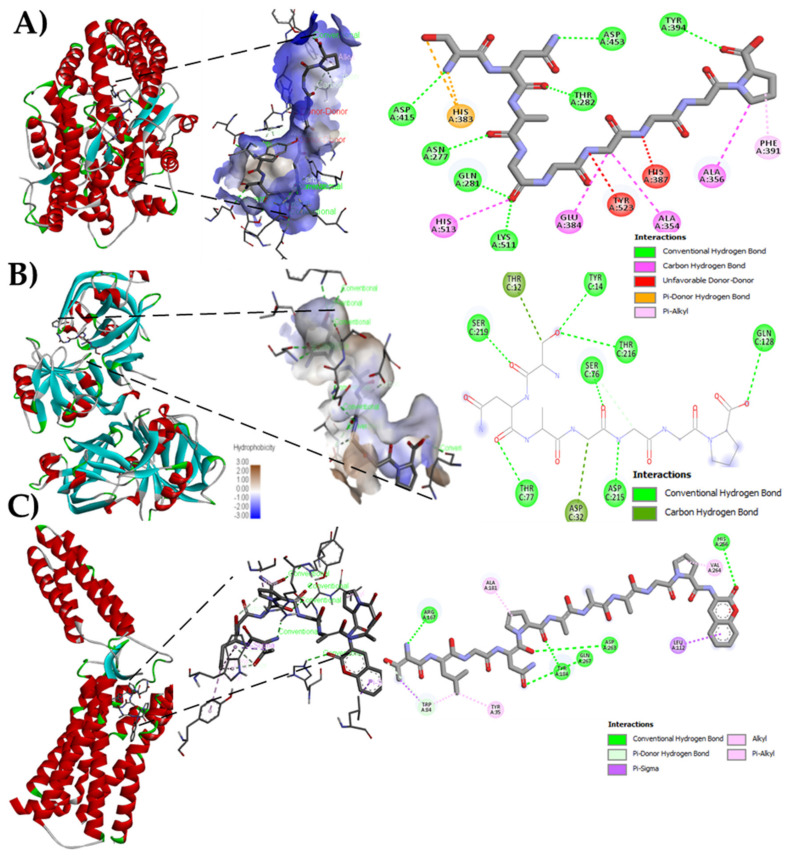
Molecular docking of native criollo peptides. (**A**) Molecular docking of peptide SNAGGGGGP with the Angiotensin Converting Enzyme (ACE) (PDB: ID 1086); (**B**) Molecular docking of peptide SNAGGGGGP with Renin (PDB: ID 2V0Z); (**C**) Molecular docking of peptide TLGNPAAAGPF with AT_1_-R (PDB: ID 4YAY).

The bioinformatics analysis by the BIOPEP-UWM database showed possible effects to control hypertension through ACE and renin inhibition. The results of the theoretical affinity of the peptides with the three molecular targets related to hypertension are shown in Table 3. The peptide with the highest theoretical affinity for ACE was SNAGGGGGP, with a value of −9.1 kcal/mol, higher than that shown by the drug Lisinopril (−7.7 kcal/mol). The potential interactions of this peptide with ACE involved seven conventional hydrogen bonds with the residues N_415_, N_277_, Q_208_, K_511_, T_282_, D_453_, and Y_394,_ starting with the nitrogen of S^1^ and ending with the carbonyl of the carboxyl group in P^9^, four carbon-hydrogen bonds (amino acid residues H_513_, E_384_, A_354_, and A_356_) from the carbonyl of G^4^ to the pyrrolidine ring of P^9^, two unfavorable donor-donor links (amino acid residues Y_523_ and H_387_), a pi-donating hydrogen double bond with hydrogen of an N^2^, and finally a pi-alkyl bond between the residue F_391_ with the pyrrolidine ring of proline^9^ (Figure 2A). Peptide SNAGGGGGP also showed the highest affinity to interact with renin (−3.9 kcal/mol), in comparison with other peptides identified in Criollo cocoa although lower to that shown by the drug Aliskiren (−7.8 kcal/mol). The interaction with peptide SNAGGGGGP implied seven conventional hydrogen bonds and two carbon bonds with the T_12_ residue and the first S^1^ carbon (Figure 2B). The Criollo cocoa peptides showed potent interaction with the AT_1_-R, having 13 of them higher affinity compared with the drug Losartan (−8.3 kcal/mol, Table 3). The highest values were obtained for peptides TLGNPAAAGPF (Figure 2C).

### 3.3. Effects of Cocoa Peptides on Blood Pressure 

**Figure 3:**
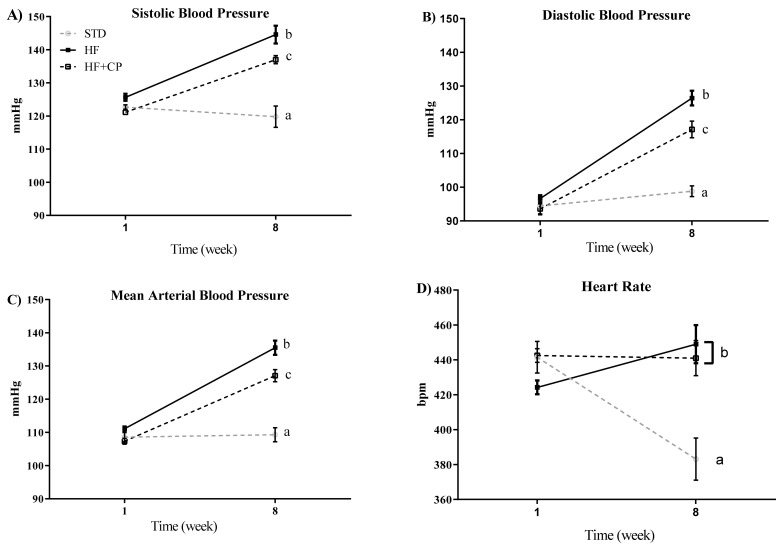
Effect of cocoa protein on blood pressure and heart rate in an obesity-induced by diet rat model. (**A**) Systolic blood pressure (SBP), (**B**) diastolic blood pressure (DBP), (**C**) mean blood pressure (MBP), and (**D**) heart rate (HR) measured by tail-cuff plethysmography at times 0 and 8 weeks. Values are expressed as mean ± SEM (*n* = 7 per group). ^a,b,c^ significantly different from STD, HF and HF + CP groups, respectively (*p* < 0.001).

The effects of cocoa peptides on the blood pressure were evaluated using an obesity-induced by diet rat model. SBP, DBP, MBP, and HR were measured in the rats of the different groups at the beginning of the study and after 8 weeks of treatment. As shown in Figure 3A–C, the intake of a HF diet significantly (*p* < 0.001) increased SBP, DBP, and MBP. However, the oral administration of CP (CP + HF group) reverted these effects, and the blood pressure significantly decreased. No effects of CP on the HR were observed, being similar in HF and CP + HF groups at the end of the assay. Only the animals fed STD diet group showed a decrease of HR levels after 8 weeks (Figure 3D).

## 4. Discussion

In the in silico study, the affinity of Criollo cocoa peptides for hypertension-related molecular targets ACE, renin, and AT_1_-R has been demonstrated. The peptide sequences obtained from Criollo CP showed several potential biological activities. According to the BIOPEP-UWM database, the potential ACE and DPP-IV inhibitory capacity, which are related to hypertension and type 2 diabetes was outstanding. Similar data were found for other vegetable proteins such as concanavalin A, B, and Canavalia from the Indonesian Jack bean (*Canavalia ensiformis*), which contained mainly ACE and DPP-IV inhibitory peptides and in a lesser extent, sequences with antithrombotic and stimulating activity [39]. In *Amaranthus hypochondriacus* (amaranth), many peptides with the potential to inhibit ACE were found [40], as it was also demonstrated for α and β subunits of chickpea (*Cicer arietinum* L.) that contained 177 and 133 ACE inhibitory peptide sequences, respectively [41]. Proteins from animal sources such as alpha-actin from tilapia (*Oreochromis* spp.) were also reported to contain bioactive peptides. Among them, 146 peptides were found to exert ACE inhibitory activity and 16 peptides demonstrated potential as antioxidants [42].

The analysis by molecular docking of the Criollo cocoa peptides showed their high affinity for ACE, renin, and AT_1_-R. These three molecular targets are the main responsible for hypertension, cardiovascular disease, and mortality [43]. Similar affinity has been previously described for peptides derived from chia proteins which sequences were TAQEPTIRF, PGLTIGDTIPNL, LSLPNYHPNPRL, LIVSPLAGRL, and IVSPLAGRL [44].

The peptides derived from *Phaseolus vulgaris* L. proteins, which sequences were GLTSK and GEGSGA, also showed a good affinity for ACE, renin, and AT_1_-R [45]. Similar results were found for peptides WG and PRY contained in potato proteins [46]. These results suggest an inhibitory effect of plant proteins derived peptides on the RAS function, thus acting reducing the hypertensive state. The in vitro analysis confirmed this potential as CPH showed ACE inhibitory ability. Similar results were observed for other vegetable proteins, such as hydrolyzed kiwicha (*Amaranthus caudatus*), and quinoa (*Chenopodium quinoa*) [47]. IC_50_ values of CPH were similar to those reported for camel whey protein hydrolyzates [48].

In the in vivo model, intragastric administration of CP in rats fed HF diet effectively lowered SBP and DBP over eight weeks of treatment. During the testing of new oral drugs, the translation of doses among animal species is essential. In this study, the key HED was obtained to translate the dose of CP from animals to humans based on the BSA. This value is used because its relationship with various biological parameters, including oxygen utilization, circulating plasma proteins, caloric expenditure, basal metabolism, blood volume, and renal function in several mammalian species. Therefore, the HED equation is recommended for converting doses from animals to humans, especially for phase I and phase II clinical trials [49]. The HED for 150 mg CP/kg/day for an adult (60 kg) is 1.46 g. To date, no data about the potential of CP to reduce blood pressure were available. Previous studies had described the blood pressure reduction with the intragastric administration of other food proteins such as Amaranth protein hydrolyzates in spontaneously hypertensive rats [13]. More recently, a study was carried out with cookies enriched with bioactive Amaranth protein hydrolysates confirmed that the baking process and the matrix of the cookies did not alter the antihypertensive effect [50]. Bean protein hydrolysate was also demonstrated to exert SBP and DBP reducing effects at doses of 20 mg/kg body weight [51].

## 5. Conclusions 

In the present study, the effect of the peptides derived from cocoa proteins against molecular targets related to high blood pressure was demonstrated by both in silico and in vitro tests. Moreover, an animal model of obesity-induced arterial hypertension was conducted to confirm the anti-hypertensive activity, demonstrating that the consumption of cocoa proteins resulted in a decrease of both SBP and DBP. Altogether, all results suggest the promising value of CP as a novel ingredient for functional foods or nutraceuticals to prevent/manage chronic disorders associated with hypertension and/or obesity. However, the translation of dose from animals to humans and the confirmation of these beneficial effects in human trials should be needed.

## Figures and Tables

**Table 1:** Concentration and percentage of T. cocoa proteins obtained without and with AcDP.

Protein Type	Fresh Cocoa Seed (Previous AcDP)		AcDP	
	mg/g	%	mg/g	%
Albumin	7.85 ^a^ ± 0.91	28.83	6.26 ^b^ ± 0.78	18.35
Globulin	0.46 ^b^ ± 0.43	1.7	2.46 ^a^ ± 0.25	7.22
Prolamin	NA		0.01 ± 0.03	0.02
Glutelin	18.92 ^b^ ± 0.85	69.46	25.38 ^a^ ± 0.52	74.41
	mg/g		mg/g	
TP pH 7.8	NA		2.94 ± 0.51	
TP pH 8	NA		3.69 ± 0.48	
TP 0.1 M NaOH	NA		21.91 ± 1.46	

AcDP (acetone dry powder). ^a,b^ indicate significantly different values (*p* < 0.05). Data are expressed as the mean of the results of two experiments realized by duplicate ± SEM. NA: not analysed; TP: total protein.

**Table 2:** Peptide sequence of Criollo cocoa obtained from in vitro hydrolysis.

Sequence	MW	Net Charge	Isoelectric Point	Hydrophobicity (Kcal/mol)	Biological Potential	Blast^®^
TLGSRTAGGCAT-GLER	1548.76	8.65	1	19.44	ACE and DPP-IV inhibitory	Arginine decarboxylase
TLHEPAGGTACLR	1324.65	7.13	0	17.09	ACE, DPP-III, DDP-IV, and renin inhibitory, antioxidant	Endochitinase 1
LTTASGGAGPFLF	1237.63	5.52	0	7.53	ACE, DPP-III, DDP-IV, and renin inhibitor, regulatory, antiamnestic, antithrombotic	Casparian strip membrane protein 1
NPGPSASGGGGATR	1184.55	10.73	1	18.76	ACE and DPP-IV inhibitor, regulatory, antiamnestic, antithrombotic, chemotactic	21 kDa seed protein
TSVSGAGGPGAGR	1072.52	10.73	1	17.31	ACE and DPP-IV inhibitor	21 kDa seed protein
LTGASPGGGAATV	1057.53	5.58	0	13.39	ACE and DPP-IV inhibitor, antioxidant, regulatory, antiamnestic, antithrombotic	Casparian strip membrane protein 1
TLGNPAAAGPF	1014.51	5.32	0	10.12	ACE, DPP-III and DDP-IV inhibitor, regulatory, antiamnestic, antithrombotic	Casparian strip membrane protein 1
VSTSGAGTTAR	1006.50	10.73	1	14.22	ACE and DPP-IV inhibitor	21 kDa seed protein
TRAGAGGGTVF	992.50	10.9	1	13.64	ACE, DPP-IV, and CaMPDE inhibitor, ubiquitin-mediated proteolysis activator	21 kDa seed protein
LTADAGLGASL	987.52	3.12	−1	12.30	ACE, DPP-III, DDP-IV, and α-Glucosidase inhibitor	Casparian strip membrane protein 1
TTRGAAGAGGAV	987.50	11.11	1	16.35	ACE, DPP-IV, and CaMPDE inhibitor, antioxidant	21 kDa seed protein
QTGGGGGGGGGGR	973.43	10.73	1	22.23	ACE and DPP-IV inhibitor	21 kDa seed protein
TLSAGGAGPGGK	971.50	9.8	1	17.05	ACE and DPP-IV inhibitor, regulatory, antiamnestic, antithrombotic	21 kDa seed protein
THPAGGGGAAR	950.46	10.73	1	18.53	ACE, DPP-III and DDP-IV inhibitor, antioxidant	21 kDa seed protein
TLSGGASGAAR	946.48	10.73	1	14.58	ACE and DPP-IV inhibitor, antioxidant	Casparian strip membrane protein 1
KMTGVVAW	890.46	9.98	1	8.92	ACE, and DPP-IV inhibitor, antioxidant	Endochitinase 1
LTTAGAAKF	878.48	9.93	1	10.89	ACE, DPP-IV, renin, and CaMPDE inhibitor, antioxidant	Casparian strip membrane protein 1
KGGPSGATGK	858.45	10.57	2	19.45	ACE and DPP-IV inhibitor, regulatory, antiamnestic, antithrombotic	Arginine decarboxylase
TTKGGSGVF	852.43	9.93	1	12.94	ACE and DPP-IV inhibitor	Maturase K
VPDGLASV	756.40	3.15	−1	11.62	ACE, DPP-III, and DPP-IV inhibitory, ubiquitin-mediated proteolysis activator	Arginine decarboxylase
SPPSGAGL	684.34	5.45	0	10.65	ACE, DPP-IV, and α-Glucosidase inhibitor	21 kDa seed protein
SNAGGGGGP	672.28	5.49	0	15.60	ACE and DPP-IV inhibitor, regulatory, antiamnestic, antithrombotic	21 kDa seed protein
LSPGGAAV	670.36	5.58	0	10.09	ACE and DPP-IV inhibitor, antioxidant, antiamnestic, antithrombotic	Vicilin
SPALNPG	654.33	5.46	0	9.89	ACE and DPP-IV inhibitor, regulatory, antiamnestic, antithrombotic	Endochitinase 1
SLTASAV	647.34	5.45	0	8.36	ACE and DPP-IV inhibitor	21 kDa seed protein
LTSAAV	560.31	5.58	0	7.90	ACE and DPP-IV inhibitor	Casparian strip membrane protein 1

ACE: angiotensin converting enzyme; DPP-III: dipeptidyl peptidase III; DPP-IV: dipeptidyl peptidase IV; Blast^®^: https://blast.ncbi.nlm.nih.gov/Blast.cgi?PROGRAM=blastp&PAGE_TYPE=BlastSearch&LINK_LOC=blasthome (accessed on 24 March 2021).

**Table 3:** Molecular docking of Criollo cocoa peptides on molecular targets related to hypertension.

Sequence	Docking Molecular		
	Affinity (kcal/mol)		
	ACE-1	Renin	AT_1_-R
TLGSRTAGGCATGLER	−5.7	−2.4	−8.8
TLHEPAGGTACLR	−6.9	−2.4	−7.6
LTTASGGAGPFLF	−6.1	−2.5	−9.5
NPGPSASGGGGATR	−6.6	−2.9	−8.5
TSVSGAGGPGAGR	−1.6	−3.0	ND
LTGASPGGGAATV	−5.8	−2.8	−10
TLGNPAAAGPF	−7.2	−3.3	−10.2
VSTSGAGTTAR	−6.5	−3.0	−9.4
TRAGAGGGTVF	−6.9	−3.3	−9.8
LTADAGLGASL	−6.4	−3.5	−9.3
TTRGAAGAGGAV	−6.4	−3.8	−9.1
QTGGGGGGGGGGR	−6.6	−2.7	−7.9
TLSAGGAGPGGK	−6.1	−2.4	−8.4
THPAGGGGAAR	−6.7	−3.2	−8.3
TLSGGASGAAR	−5.8	−2.1	−8.3
KMTGVVAW	−6.5	−3.4	−9.0
LTTAGAAKF	−5.4	−2.5	−8.3
KGGPSGATGK	−5.3	−2.8	−8.1
TTKGGSGVF	−5.5	−2.2	−8.4
VPDGLASV	−6.6	−4.0	−8.9
SPPSGAGL	−7.6	−3.3	−8.2
SNAGGGGGP	9.1	−3.9	−7.8
LSPGGAAV	−8.6	−3.2	−8.5
SPALNPG	−6.8	−4.0	−9.0
SLTASAV	−6.3	−3.9	−8.1
LTSAAV	−5.2	−3.1	−7.0
Lisopril	−7.7	-	-
Aliskiren	-	−7.8	-
Losartan	-	-	−8.3

ACE: Angiotensin converting enzyme. ND: Not docking. LTGASPGGGAATV, TRAGAGGGTVF, LTTASGGAGPFLF, and VSTSGAGTTAR with affinity values of −10.2, −10, −9.8, −9.5, and −9.4 kcal/mol, respectively. Confirmation of the ACE inhibitory activity was performed by an in vitro analysis, obtaining an IC_50_ value of 0.49 mg of CPH/mL.

## Data Availability

Not applicable.
